# Physician’s Perceptions of Interprofessional Collaboration in Clinical Training Hospitals in Northeastern Japan

**DOI:** 10.4021/jocmr1474w

**Published:** 2013-08-05

**Authors:** Sachiko Minamizono, Hitoshi Hasegawa, Naoko Hasunuma, Yoshihiro Kaneko, Yutaka Motohashi, Yuji Inoue

**Affiliations:** a Department of Health Care Policy, Akita University Graduate School of Medicine, 1-1-1 Hondo, Akita, Japan; b Department of Community Medicine and Primary Care Development, Akita University School of Medicine, 1-1-1 Hondo, Akita, Japan; c Department of Public Health, Akita University Graduate School of Medicine, 1-1-1 Hondo, Akita, Japan

**Keywords:** Physician’s role, Cooperative behavior, Interprofessional collaboration, Interdepartmental relations, Patient-centered care, Professional practice

## Abstract

**Background:**

Effective and efficient interprofessional collaboration (IPC) is needed between departments in a healthcare setting. Although Japanese physicians are expected to provide leadership in IPC, it has been suggested that their perception of IPC is more negative than among other healthcare professionals. The purpose of this study was to clarify Japanese physician’s perceptions of IPC and what factors influenced their views.

**Methods:**

This cross-sectional study surveyed 732 medical doctors at a university hospital and six foundation hospitals in a prefecture located in Tohoku district, northeastern Japan. Those hospitals were approved for delivery of postgraduate clinical training. Physician’s perceptions of IPC were investigated for three items, namely providing patient-centered care, preventing medical accidents, and improving the quality of medical care. A total of 409 doctors who were engaged in clinical practice, responded adequately to the survey. Factors associated with negative perceptions towards IPC among physicians were analyzed using a logistic regression model.

**Results:**

The proportion of negative perceptions of IPC for providing patient-centered care, preventing medical accidents, and improving the quality of medical care were 41.1%, 34.0% and 33.7%, respectively. Negative perceptions of IPC for providing patient-centered care were associated with older age (50 + years; odds ratio (OR): 2.73; 95% confidence interval (CI): 1.11 - 6.68) and a lower frequency of interprofessional meetings (no meetings; OR: 2.95; 95%CI: 1.43 - 6.08). Negative perceptions of IPC for preventing medical accidents were associated with a lower frequency of interprofessional meetings (no meetings, OR: 3.23; 95%CI: 1.58 - 6.62). Negative perceptions of IPC for improving the quality of medical care were associated with middle age (40 - 49 years, OR: 2.93; 95%CI: 1.20 - 7.12) and a lower frequency of interprofessional meetings (no meetings; OR: 2.75; 95%CI: 1.34 - 5.66).

**Conclusions:**

Physician’s negative perceptions of IPC in our study were associated with age and a lower frequency of interprofessional meetings. Our findings suggest that effective regular interprofessional meetings serve to share information about patients, and to allow physicians to understand each other better, which should have a positive impact on the quality of patient-centered care.

## Introduction

Interprofessional collaboration (IPC) in medicine is a process in which different professional groups work together to ensure a positive impact on each other and on patient care. The World Health Organization (WHO) Framework for Action on Interprofessional Education and Collaborative Practice reported evidence for the benefits of such practice [1]. These benefits included improved use of specialist resources, better patient care and safety, and improved health outcomes for patients with chronic disease. They also led to a reduction in complications and length of hospital stay, and reduced clinical error rates and mortality rates.

In order to realize effective IPC, several factors are taken into consideration; good communication patterns, appropriate power dynamics, and understanding of roles and responsibilities of healthcare staff [2-7]. For example, a lack of clarity on professional and functional roles, responsibilities and accountability can lead to a breakdown in communication that may have a direct impact on patients [3, 4, 8-10]. Analysis of teamwork and communication in clinical teams identified recurrent complicated problems caused by hierarchical and cultural barriers among healthcare workers [11].

Unfortunately, IPC has not been introduced effectively in Japan, possibly as a result of a lack of communication among healthcare staff, strong hierarchical barriers, and a lack of understanding of healthcare staff about their own and their colleagues’ roles and responsibilities. It has been suggested that the level of awareness of interprofessional practice among physicians was lower than those of other healthcare professionals [12-14]. Additionally, Japanese physicians have been institutionally expected to play a leadership role in healthcare settings [15]. As a consequence, physicians tend to adhere to hierarchical human relationships in healthcare settings in Japan.

In order to develop effective IPC in Japan, it is necessary for physicians to have a better understanding of IPC and to develop communication skills with healthcare staff. Furthermore, a physician’s perception of IPC in clinical settings is also an important factor to be considered, and has not been sufficiently investigated. Thus, the purpose of the present study was to clarify what factors are associated with negative perceptions of IPC among physicians working in clinical teaching hospitals in Japan.

## Methods

### Participants

A cross-sectional survey on physician’s perceptions of IPC was conducted from February to March 2011, using self-administered anonymous questionnaires. The participants were full-time physicians who had completed a 2-year postgraduate residency program or had a minimum of 2 years’ working experience as a medical doctor. The participants were working in a university hospital or six foundation hospitals in a prefecture in Tohoku district, in northeastern Japan. The seven hospitals were approved by the Ministry of Health, Labor and Welfare to provide postgraduate clinical training. Additionally, they had 10 or more residents, no fewer than 70 physicians, and more than 450 beds.

A questionnaire was delivered to 732 physicians. A total of 431 (58.9%) responded adequately to the questionnaire. Each questionnaire, sealed in an envelope, was collected from a respondent by site staff and sent directly to the authors. A total of 409 physicians (54.4%) gave complete answers in the questionnaire.

### Questionnaire

The questionnaire obtained data on demographic variables, frequency of interprofessional meetings and perceptions of IPC. A number of measurement scales for interprofessional collaboration have been developed for one health professional group, namely nurses. A few measurement scales for multiple health professional groups have been developed recently. These scales were designed to assess actual teamwork within healthcare settings, or concentrated on interprofessional education (IPE) [16-18]. In this study, we required items related to physician’s perceptions of IPC, not actual IPC assessment and IPE assessment. Therefore, we referred to the benefits of IPC as specified by the WHO, and Heinemannn’s Attitudes Toward Health Care Teams, which consists of two components: Quality of Care/Process and Physician Centrality in an actual healthcare team [16]. The items in our questionnaire related to the physician’s perceptions of IPC regarding three aspects: providing patient-centered care, preventing medical accidents, and improving the quality of medical care. These aspects were in line with the WHO framework for action on IPE and collaborative practice, which cites research evidence for the benefits of both [1].

We formulated the following three questions on perceptions of IPC: ‘Do you think IPC at your work site is useful for providing patient-centered care?’; ‘Do you think IPC at your work site is useful for preventing medical accidents?’; and ‘Do you think IPC at your work site is useful for improving quality of medical care?’. Possible answers were: “Strongly agree”, “Agree”, “Disagree”, and “Strongly disagree”.

### Statistical analysis

Data on the perceptions of the three aspects of IPC were classified into two groups of positive and negative: strongly agree and agree were classified as indicators of positive perceptions, disagree and strongly disagree were classified as negative perceptions. Logistic regression analyses were used to identify factors contributing to negative perceptions of the physicians towards IPC. For all statistical tests, a significant difference was defined as P < 0.05. Statistical analysis was conducted using SPSS version 17.0 software (SPSS Inc., Chicago, IL, USA).

### Ethics

This survey was approved by the Ethics Committee of Akita University Faculty of Medicine. To protect the privacy of respondents, it was clearly stated on the questionnaire that the completed questionnaire would only be seen by the researchers and that the results would be anonymized.

## Results

The characteristics of the respondents are presented in Table 1. Of the 409 physicians (346 male, 63 female), 68.9% were aged 30 - 49 years. Data on the frequency of interprofessional meetings indicated that 42.8% of physicians reported having meetings at least once a week and 10.3% reported never having meetings.

**Table 1:** Characteristics of Study Participants

	Physicians
	N = 409
	%
Gender	
male	84.6
female	15.4
Age (years)	
26 - 29	9.8
30 - 39	36.4
40 - 49	32.5
50 +	21.3
Type of hospital	
University hospital	52.8
Foundation hospital	47.2
Type of department	
Internal medicine	16.1
Surgical	24.9
others	58.9
Frequency of interprofessional meetings	
once a week +	42.8
once a month	26.9
few times a year	20.0
never	10.3

The overall proportion of negative perceptions of IPC for providing patient-centered care, preventing medical accidents and improving quality of medical care were 41.1%, 34.0% and 33.7%, respectively. Table 2 presents the results of logistic regression analyses to identify factors associated with negative perceptions of IPC among physicians. A negative perception of IPC for providing patient-centered care was associated with older age (50 + years; odds ratio (OR): 2.73; 95% confidence interval (CI): 1.11 - 6.68) and lower frequency of interprofessional meetings (no meetings; OR: 2.95; 95%CI: 1.43 - 6.08). A negative perception of IPC for preventing medical accidents was associated with lower frequency of interprofessional meetings (no meetings; OR: 3.23; 95%CI: 1.58 - 6.62). A negative perception of IPC for improving quality of medical care was associated with middle age (40 - 49 years; OR: 2.93; 95%CI: 1.20 - 7.12) and a lower frequency of interprofessional meetings (no meetings; OR: 2.75, 95%CI: 1.34 - 5.66). Gender and specialty had no significant effect on perceptions of IPC.

**Table 2:** Multiple Logistic Regression Analysis of Factors Associated With Negative Perceptions of IPC Among Physicians

	Negative perceptions of IPC		
	Providing patient-centered care	Preventing medical accidents	Improving quality of medical care
	
	Adjusted OR (95%CI)	Adjusted OR (95%CI)	Adjusted OR (95%CI)
Gender			
male	1.00	1.00	1.00
female	1.55 (0.85 - 2.82)	1.16 (0.63 - 2.15)	1.05 (0.56 - 1.98)
Age (years)			
26 - 29	1.00	1.00	1.00
30 - 39	3.23 (1.40 - 7.44)	1.38 (0.64 - 2.98)	2.37 (0.98 - 5.70)
40 - 49	2.88 (1.23 - 6.73)	1.34 (0.61 - 2.93)	2.93 (1.20 - 7.12)
50 +	2.73 (1.11 - 6.68)	0.82 (0.35 - 1.93)	1.80 (0.69 - 4.59)
Type of department			
internal medicine	1.00	1.00	1.00
surgical medicine	1.30 (0.66 - 2.52)	1.73 (0.85 - 3.53)	1.88 (0.93 - 3.79)
others	1.08 (0.60 - 1.92)	1.50 (0.80 - 2.81)	1.23 (0.66 - 2.28)
Type of hospital			
University hospital	1.00	1.00	1.00
Foundation hospital	0.86 (0.56 - 1.32)	0.81 (0.52 - 1.27)	0.83 (0.53 - 1.30)
Frequency of interprofessional meetings			
once a week +	1.00	1.00	1.00
once a month	1.53 (0.91 - 2.55)	1.20 (0.70 - 2.06)	1.08 (0.62 - 1.88)
few times a year	1.77 (1.02 - 3.06)	1.89 (1.08 - 3.33)	1.91 (1.09 - 3.35)
never	2.95 (1.43 - 6.08)	3.23 (1.58 - 6.62)	2.75 (1.34 - 5.66)

Multiple logistic regression models were adjusted for all items in the table. IPC: Interprofessional collaboration; OR: Odds ratio; CI: Confidence interval.

## Discussion

Factors that contributed to a negative perception of IPC were age and a lower frequency of interprofessional meetings. We considered that older age was likely to be associated with negative perceptions of IPC. It is well recognized that the balance of power in hospitals lies with older, more established clinical professionals than with other healthcare professionals [19]. This study indicated that physicians with established positions in hospitals seemed to have less motivation for IPC.

In medical schools in Japan, a model core curriculum was proposed by the government in 2001 [20-22], which included “communication at work” and “team behavior” (effectively IPC in healthcare) as basic issues. Additionally, the importance of team behavior was included as a general educational objective. After the introduction of the medical curriculum in 2001, it has been speculated that perceptions of IPC among young doctors have improved. Our study appears to confirm that perceptions of IPC were more positive in younger doctors compared with older physicians. Additionally, the concept of patient-centered healthcare has become widely used only in recent years, after the phrase “patient-centered medicine” was introduced in the model core curriculum in 2001. In 2010, the importance of team behavior in patient-centered healthcare was also incorporated into a general educational objective to respond to greater social expectation. Our finding that there was a poor understanding of this aspect of IPC among older physicians can be related to their different educational background, but this also suggests a lack of recognition during their career about professional responsibility for IPC in healthcare. Therefore, it was indicated that promoting IPC is necessary for sustaining awareness of IPC in postgraduate medical education and continuing professional development.

An association between frequency of interprofessional meetings and perceptions of IPC was observed. Some studies reported that interprofessional meetings improved some health outcomes of patients [23, 24]. Schmidt et al reported that monthly multidisciplinary team meetings improved psychotropic drug prescribing practices in nursing homes. Wilson et al. reported that video conferencing compared with audio conferencing decreased the number of case conferences per patient and shortened the length of treatment. Purtilo and Haddad stressed that verbal communication, such as in meetings, was essential for the patient and to health professional relationships [25]. Regular meetings of multiprofessional teams help to enforce verbal communication and to activate collaboration. Although we only performed a cross-sectional study, the findings were consistent with these reports, and stressed the importance of having regular face-to-face meetings. Without interprofessional meetings, health professionals tend to carry on working without realizing the advantages of IPC [26]. Regarding the frequency of interprofessional meetings, it was reported that meetings at least once a month were considered most appropriate for improving communication among healthcare professionals [23], and this is also supported in this study.

A recent study reported that female students had a more positive attitude to teamwork than male students among medical and nursing staff [27]. However, our study did not reveal any relationship between gender and perceptions of IPC among physicians. In spite of many study interventions in various fields [1, 2], there are few reports confirming any association between IPC and specific specialties. In the present study, even after adjusting for the frequency of meetings and type of hospital, there was no significant association between specialty and perceptions of IPC. This suggests that the importance and necessity of IPC has become recognized among physicians in all types of clinical setting.

To promote IPC and interprofessional education in Japan, postgraduate medical education and continuing professional development is important, in addition to undergraduate medical education. A number of factors, including specific personal power, status, professional socialization and decision-making responsibilities are barriers to the engagement of physicians in collaborative practice [7]. Therefore, physicians are expected to make considerable efforts to overcome these barriers in order to improve IPC and patient care.

We hope that our findings will be useful to raise the awareness of IPC in hospital doctors and to improve communication among healthcare staff. Further research will be required to evaluate the effectiveness of IPC and to follow up the changing awareness of healthcare staff and residents towards IPC.

### Study limitations

Our study has some limitations. First, perceptions of three aspects of interprofessional collaboration were assessed with only one question each. Cronbach’s alpha for the three items was 0.905, revealing a high rate of internal consistency. Second, the targeted hospitals were located in one prefecture of Japan. Consequently, it is difficult to generalize our results. Third, the use of self-administered questionnaires might have incurred selection bias, as respondents might have more positive perceptions than non-respondents. The highest percentages of unanswered questions were as follows: age, 0.94%; and type of department 1.17%. Of the 22 respondents who did not complete all the questionnaire, 80.0% were male, 50.0% were aged in their 30’s, 68.2% were working at a university hospital, and 47.1% were working as surgeons. There were no statistically significant differences between complete and incomplete responders with respect to perceptions of IPC. Finally, we could not determine causal relationships because of the cross-sectional study design.

### Conclusions

Negative perceptions of IPC among physicians working in clinical training hospitals in northeastern Japan were associated with older age and a lower frequency of interprofessional meetings.
